# Genetic insights into fetal growth and measures of glycaemic regulation and adiposity in adulthood: a family-based study

**DOI:** 10.1186/s12881-018-0718-2

**Published:** 2018-12-04

**Authors:** Mette Hollensted, Claus T. Ekstrøm, Oluf Pedersen, Hans Eiberg, Torben Hansen, Anette Prior Gjesing

**Affiliations:** 1 Section of Metabolic Genetics, The Novo Nordisk Foundation Center for Basic Metabolic Research, Faculty of Health and Medical Sciences, University of Copenhagen, Blegdamsvej 3B, DK-2200 Copenhagen, Denmark; 2 The Danish Diabetes Academy, Odense, Denmark; 3 Section of Biostatistics, Department of Public Health, Faculty of Health and Medical Sciences, University of Copenhagen, Copenhagen, Denmark; 4 Department of Cellular and Molecular Medicine, Faculty of Health and Medical Sciences, University of Copenhagen, Copenhagen, Denmark; 5 Faculty of Health Sciences, University of Southern Denmark, Odense, Denmark

**Keywords:** Birth weight, Body composition, Fetal growth, Genetic association, Genetic correlation, Heritability, Insulin secretion, Insulin sensitivity

## Abstract

**Background:**

The genetics of fetal insulin release and/or action have been suggested to affect fetal growth, adult insulin resistance and adult body composition. The genetic correlation between body composition at birth versus glycaemic regulation and body composition in adulthood have, however, not been well studied. We therefore aimed to investigate these genetic correlations in a family-based cohort.

**Methods:**

A Danish family cohort of 434 individuals underwent an oral glucose tolerance test with subsequent calculation of surrogate measures of serum insulin response and insulin sensitivity. Measures of fetal growth were retrieved from midwife journals. Heritability and genetic correlations were estimated using a variance component model.

**Results:**

A high heritability of 0.80 was found for birth weight, whereas ponderal index had a heritability of 0.46. Adult insulin sensitivity measured as Matsuda index was genetically correlated with both birth weight and ponderal index (ρG = 0.36 (95% CI: 0.03; 0.69) and ρG = 0.52 (95% CI, 0.15; 0.89), respectively). Only birth weight showed a significant genetic correlation with adult weight (ρG = 0.38 (95% CI: 0.09; 0.67)) whereas only ponderal index was genetically inversely correlated with fasting insulin (ρG = - 0.47 (95% CI: - 0.86; - 0.08) and area under the curve for insulin release during the oral glucose tolerance test (ρG = - 0.66 (95% CI: - 1.13; - 0.19)).

Individual as well as combined adjustment for 45 selected birth weight, obesity and type 2 diabetes susceptibility gene variants did not affect the correlations.

**Conclusions:**

The genetics of both birth weight and ponderal index appear to be under the same genetic influence as adult insulin resistance. Furthermore, ponderal index and adult insulin release seem to be partly shared, as well as the genetics of birth weight and adult weight.

Word count abstract: 281.

**Electronic supplementary material:**

The online version of this article (10.1186/s12881-018-0718-2) contains supplementary material, which is available to authorized users.

## Introduction

Low birth weight associates with adult insulin resistance, hypertension, coronary-artery disease and type 2 diabetes mellitus (T2DM) [1, 2], possibly due to maternal malnutrition [3, 4] or an insulin-resistant/-deficient genotype of the foetus [5]. The latter hypothesis, known as the fetal insulin hypothesis, suggests that genetically induced insulin resistance or altered insulin secretion causes impaired insulin-mediated growth in the foetus and insulin resistance/insulin deficiency in adult life [5]. Birth weight has also been found to display a positive phenotypical correlation with measures of adult body mass index (BMI) [6–8]. Although environmental factors, such as intrauterine conditions, have been shown to influence this correlation [8], it has also been hypothesized that the genetic background of birth weight and adult BMI may be partly overlapping [9, 10]. Yet, the ~ 100 obesity susceptibility loci identified so far [11] appear to have a limited influence on birth weight [12, 13], despite the identification of genetic correlations between birth weight and traits related to adult obesity, including BMI, waist and waist-hip ratio using linkage disequilibrium (LD) score regression [14]. Out of 60 genetic loci associating with birth weight in a genome-wide association study (GWAS) meta-analysis among European individuals (*n* = 153,781) [14], several, including *ADCY5*, *CDKAL1, HHEX-IDE* and *ANK1,* are also known to associate with diabetes [14–17], confirming the shared genetics between birth weight and glucose regulation. In the same study, the link between birth weight and T2DM was further demonstrated when using LD score regression, as birth weight was inversely genetically correlated with T2DM [14]. LD score regression is, however, based on summary statistics from previous GWAS, and the effect of rare genetic variation is not considered. Neither do these large-scale studies include detailed phenotypes related to adult glycaemic regulation and body composition.

The primary aim of the current study was therefore to assess the degree of genetic correlations between fetal body composition and measures of insulin sensitivity in a Danish family population with detailed phenotypes using a variance component model, thereby considering the effect of both common and rare genetic variation. Secondly, we aimed to assess the genetic correlations between fetal body composition and measures of adult body composition, and finally, the genetic correlation between fetal body composition and adult levels of insulin secretion. For significant genetic correlations, we aimed to examine whether single nucleotide polymorphisms (SNPs) known to associate with birth weight, adult risk of obesity or adult risk of T2DM may explain any shared genetics between the investigated traits. However, as the presence of a significant genetic heritability of included traits is a prerequisite for genetic correlation analysis, heritability estimates were made for all included traits.

## Methods

### Participants

The study population consisted of 533 individuals from 93 Danish families recruited between July 1993 and April 1999. In each family, one parent was suffering from verified T2DM according to World Health Organization 1999 criteria [18], while the other parent had no known form of diabetes. Patients were recruited through the outpatient clinic at Steno Diabetes Center (Gentofte, Denmark) or through a family study performed at the University of Copenhagen (Copenhagen, Denmark). At diagnosis, probands (*n* = 93) were ≥ 40 years of age and had no known family history of type 1 diabetes. All family members, i.e. spouses, offspring, and other relatives, were invited to participate and had an oral glucose tolerance test (OGTT) performed. In the current study, only individuals from families with clinical information on a trio or more family members were included (*n* = 434). These individuals came from 62 families, each comprising 3–12 individuals. In total, 31 individuals receiving diabetes treatment were removed from analyses involving measures of glucose metabolism.

### Clinical examination

Fetal growth measures were retrieved from midwife’s journals in the local Danish Provincial Archive, and ponderal index was calculated as weight (kg)/length (m)^3^. When calculating measures of adult body composition and insulin secretion and sensitivity, individuals as young as 14 years were included. Although these measures therefore include information obtained from youths, the far majority of examined individuals were adults (≥ 18 years of age). For this reason, as well as for simplicity, these measures will be termed adult measures in the following. Adult height, weight, waist and hip circumference were measured while wearing light indoor clothes and no shoes. BMI was calculated as weight in kilos divided by the height in meters squared (kg/m^2^), while waist-hip ratio was calculated as waist circumference (cm)/hip circumference (cm). Body fat mass and fat free mass were determined by bioelectrical impedance [19]. Venous blood samples were drawn after a 12-h overnight fast with measurements of serum insulin and blood glucose. In individuals without known T2DM, a standardized OGTT defined by the World Health Organization was performed with 15 time points from *t* = 0 to *t* = 240 min. Details of the biochemical analyses of glucose and insulin concentrations have been described previously [20]. Clinical characteristics of the study population are provided in Table 1, and an overview of the family relations is provided in Table 2.

**Table 1:** Phenotypic characterization of the study population

Family relation	Probands (*n* = 58)		Offspring (*n* = 249)		Other relatives (*n* = 79)		Spouses (*n* = 48)	
Trait	Mean (SD)	Range	Mean (SD)	Range	Mean (SD)	Range	Mean (SD)	Range
Body composition at birth								
Sex (men/women)	34/24	NA	118/131	NA	38/41	NA	12/36	NA
Birth weight (g)	3495 (510.9)	2200, 4000	3573 (582.1)	1950, 5500	3622 (467.3)	2300, 4400	3466 (491.8)	1950, 4500
Birth length (cm)	50.8 (2.2)	46.0, 54.0	52.3 (2.2)	45.0, 59.0	52.3 (2.3)	47.0, 59.0	50.7 (2.4)	42.0, 54.0
Ponderal index	26.5 (3.6)	21.3, 36.2	24.9 (2.8)	12.7, 33.6	25.4 (3.1)	19.1, 36.2	26.6 (2.7)	21.3, 32.0
Body composition in adulthood								
Age (years)	67.8 (8.2)	43.3, 86.1	40.6 (9.0)	14.9, 73.0	48.9 (16.9)	19.7, 83.6	66.1 (8.2)	42.5, 88.2
Weight (kg)	88.2 (17.7)	56.5, 153.0	78.6 (16.2)	47.5, 134.2	80.3 (16.9)	48.0, 124.0	78.0 (12.5)	57.3, 110.0
Height (cm)	169.0 (10.7)	151.0, 191.5	173.1 (8.6)	154.0, 200.0	169.8 (9.3)	147.0, 192.0	165 (6.9)	152.0, 179.0
Waist (cm)	101.1 (14.1)	79.0, 150.0	86.3 (13.9)	61.0, 128.0	90.9 (14.4)	63.0, 126.0	92.0 (11.4)	72.0, 118.0
Hip (cm)	105.9 (10.5)	91.0, 155.0	99.6 (8.89)	76.0, 132.0	101.9 (11.4)	81.0, 137.0	104.1 (9.8)	87.0, 131.0
Waist-hip ratio	0.95 (0.1)	0.79, 1.19	0.87 (0.10)	0.67, 1.17	0.89 (0.1)	0.68, 1.10	0.88 (0.08)	0.71, 1.09
BMI (kg/m^2^)	30.8 (5.3)	22.3, 48.8	26.2 (4.85)	16.9, 43.1	27.8 (5.3)	17.9, 43.4	28.8 (5.2)	20.3, 40.8
Fat free mass (kg)	56.5 (13.0)	36.3, 84.2	52.2 (10.8)	30.3, 86.0	53.7 (12.6)	35.8, 85.6	46.5 (7.6)	32.8, 66.6
Fat mass (kg)	32.6 (11.2)	14.2, 79.5	25.6 (11.1)	3.76, 58.8	30.4 (11.6)	5.91, 60.6	30.2 (11.3)	12.0, 58.2
Glyceamic regulation in adulthood								
	Probands (*n* = 28)		Offspring (*n* = 246)		Other relatives (*n* = 77)		Spouses (*n* = 47)	
Fasting serum insulin (pmol/l)	66.7 (37.3)	18.0, 160.0	43.0 (31.5)	5.67, 210.3	55.1 (40.4)	15.7, 253.0	52.2 (41.7)	18.3, 267.3
HOMA-B (%)	388.7 (315.8)	58.5, 1090	520.6 (361.5)	75.0, 3182	530.2 (306.9)	73.6, 1853	590.5 (444.5)	170.7, 2839
BIGTT-AIR	998.0 (874.6)	140.9, 1889	2608.0 (1364)	82.7, 9238	2705 (2032)	1261, 14,000	4593 (10884)	1244, 66,560
Insulinogenic index	26.49 (25.2)	7.20, 63.5	134.3 (422.2)	8.35, 5970	101.8 (117.8)	4.91, 815.2	95.5 (75.0)	15.3, 368.3
AUC insulin 4 h (pmol/l*min)	36,600 (30016)	14,350, 77,980	45,380 (32892)	12,120, 223,400	54,370 (30367)	8060, 125,800	68,500 (56029)	21,790, 329,300
Insulin sensitivity								
HOMA-IR	22.2 (14.7)	4.98, 57.9	10.8 (9.47)	0.95, 57.5	15.9 (20.1)	3.52, 156.3	13.1 (11.1)	3.90, 64.0
Matsuda insulin sensitivity index	10.5 (7.3)	2.35, 26.3	10.5 (6.08)	1.50, 38.3	8.81 (5.6)	1.10, 24.7	9.26 (7.43)	0.96, 39.0

Data is presented as means, SD and range for the four different types of family relation (probands, offspring, other relatives and spouses). AUC: area under the curve, BIGTT-AIR: index for acute insulin response, HOMA-B: HOMA for beta cell function, HOMA-IR: HOMA for insulin resistance

**Table 2:** Relationships of relative pairs included in analysis

Relationship	*n*
Parent-offspring	173
Siblings	387
Avuncular	82
Half-siblings	16
Third-degree	83
Fourth-degree	56
Unrelated	36

### Variants known to associate with birth weight, BMI and T2DM

Genotyping was performed using either the MetaboChip [21] or the Illumina HumanCoreExome Beadchip. Both types of genotyping were done on an Illumina HiScan (Illumina, San Diego, CA, USA), and the Genotyping module (version 1.9.4) of GenomeStudio software (version 2011.1, Illumina) was used to call MetaboChip-derived genotypes. Quality control of HumanCoreExome genotypes was performed using Illumina GenCall Data Analysis Software.

Fifteen SNPs or proxies (*r*^2^ > 0.90, D’ = 1.000) known to exert the largest effect on birth weight, adult BMI and T2DM risk among Caucasians were selected based on availability (Additional file 1).

Individuals and SNPs with a low call rate (< 99%) were excluded from our analyses. In addition, individuals were excluded, if their calculated kinship matrix (as based on genotypes) did not correspond to their questionnaire information. This was assessed using an Identical-By-Descent plot in Plink [22].

### Calculation of surrogate measures of insulin sensitivity and insulin release

Based on the OGTT, we calculated surrogate measures of pancreatic beta-cell insulin secretion (HOMA-index for pancreatic beta cell function (HOMA-B), the index for acute serum insulin response based on the OGTT (BIGTT-AIR), the insulinogenic index, and the area under the curve (AUC) for insulin) as well as surrogate measures of insulin resistance (HOMA-index for insulin resistance (HOMA-IR) and insulin sensitivity (Matsuda insulin sensitivity index). HOMA-IR is based on measures in the fasted state in contrast to Matsuda insulin sensitivity index, which includes insulin sensitivity in the glucose-stimulated state. AUC for serum insulin was calculated using the trapezoid method, and calculations of the remaining indices can be found in Additional file 2.

### Statistical analyses

Heritabilities and genetic correlations were calculated using a variance component model in SOLAR software package, version 4.2.0 [23, 24]. All analyses were adjusted for sex. Heritability estimates were calculated using the ACE model, which estimates the genetic effect (A), the unique environment effect (E) as well as the common environmental effect, i.e. the household effect (C). In the current study, the common shared environment (C) was defined by households. These effects are expressed as estimated percentages of the total phenotypic variance (P) of a given trait and are termed *h*^*2*^ (the additive genetic effect), *c*^*2*^ (the effect of the shared environment) and *e*^*2*^ (the effect of the unique environment). The heritability of fasting insulin has previously been reported in our study population [20, 25].

Phenotypic and genetic correlations were measured as Pearson’s phenotypic correlation coefficient (ρP) and Pearson’s genetic correlation coefficient (ρG), respectively. Calculations were performed using SOLAR which tests whether Pearson’s phenotypic correlation coefficient is significantly different from 0, and whether Pearson’s genetic correlation coefficient is significantly different from 0 or 1. All genetic correlations performed on adult phenotypes were estimated using adult age-adjusted traits. Furthermore, correlations involving traits related to glycaemic control were performed using BMI-adjusted glycaemic traits. In case of non-normally distributed traits or residuals, an inverse normal transformation was applied, as the kurtosis would otherwise be too high.

As a means to assess the genetic overlap between fetal and adult traits ascribed to selected high effect-sized SNPs known to associate with birth weight, adult risk of obesity or adult risk of T2DM, these SNPs were included in the bivariate SOLAR model both as individual covariates and as a combined list of covariates.

Heritability analyses did not undergo correction for multiple testing and neither did analyses based on our primary hypothesis of a genetic correlation between fetal body composition and surrogate measures of adult insulin sensitivity. Thus, for these analyses a *p*-value < 0.05 was considered statistically significant. However, investigation of our secondary hypothesis of the genetic correlations between fetal body composition and measures of body composition and insulin secretion in adulthood was corrected for multiple testing. Traits estimating insulin secretion (HOMA-B, BIGTT-AIR, insulinogenic index and AUC insulin 4 h) are highly correlated, as they are estimated from the same input data. This is also the case for measurements of adult body composition (weight, height, waist, waist-to-hip ratio, BMI and fat mass). Thus, a *p*-value < 0.025 (0.05/2) was considered statistically significant for genetic correlations between fetal body composition and adult body composition as well as between fetal body composition and adult insulin secretion as we are correcting for these two overall physiological traits (adult body composition and insulin secretion).

## Results

### Estimates of heritability

A high genetic heritability of 0.80 (95% CI: 0.37; 1.23) was found for birth weight, whereas ponderal index had a heritability of 0.46 (95% CI: 0.01; 0.91). Upon adjustment for shared environment, the genetic heritability for birth length was non-significant (Table 3).

**Table 3:** Heritability estimates

Body composition measured at birth				
Trait	*n*	*h*^2^ (95% CI)	*c*^*2*^ (95% CI)	*e*^2^ (95% CI)
Birth weight (g)	297	0.80 (0.37; 1.23)*	0.05 (−0.20; 0.30)	0.15 (−0.09; 0.39)
Ponderal index	296	0.46 (0.01; 0.91)*	0.02 (− 0.18; 0.22)	0.53 (0.22; 0.84)*
Birth length (cm)	296	0.15 (−0.54; 0.84)	0.17 (−0.18; 0.52)	0.67 (0.30; 1.04)*
Adult body composition				
Weight (kg)	406	0.35 (0.04; 0.66)*	0.01 (−0.15; 0.17)	0.64 (0.42; 0.86)*
Height (cm)	406	0.30 (0.01; 0.59)*	0.32 (0.12; 0.52)*	0.39 (0.21; 0.57)*
Waist (cm)	398	0.28 (−0.01; 0.57)*	0.03 (−0.13; 0.19)	0.69 (0.49; 0.89)*
Waist-hip ratio	397	0.34 (0.14; 0.54)*	0 (0)	0.66 (0.46; 0.86)*
BMI (kg/m^2^)	406	0.32 (0.03; 0.61)*	0.07 (−0.07; 0.21)	0.61 (0.39; 0.83)*
Fat mass (kg)	338	0.50 (0.26; 0.74)*	0 (0)	0.50 (0.26; 0.74)*
Fat free mass (kg)	338	0.09 (−0.52; 0.70)	0.17 (−0.16; 0.50)	0.74 (0.39; 1.09)*
Adult measures of glycaemic control				
Insulin secretion				
HOMA-B (%)	367	0.27 (−0.06; 0.60)*	0.02 (−0.16; 0.20)	0.71 (0.38; 1.04)*
BIGTT-AIR	312	0.31 (0.09; 0.53)*	0 (0)	0.69 (0.47; 0.91)*
Insulinogenic index	318	0.25 (0.03; 0.47)*	0 (0)	0.75 (0.53; 0.97)*
AUC insulin 4 h (pmol/l*min)	302	0.25 (1.01; 0.49)*	0 (0)	0.75 (0.51; 0.99)*
Insulin sensitivity				
HOMA-IR	367	0.37 (0.12; 0.62)*	0 (0)	0.63 (0.38; 0.88)*
Matsuda insulin sensitivity index	367	0.31 (0.07; 0.55)*	0 (0)	0.69 (0.45; 0.93)*

Heritabillity estimates were calculated using the ACE model. All heritability estimates were adjusted for sex, and heritability estimates of adult measures were additionally adjusted for age

BIGTT-AIR: index for acute insulin response, *c*^*2*^ = common environmental effect, *e*^*2*^ = unique environmental effect, *h*^*2*^ = additive genetic effect. *Heritability estimate significantly different from zero (*p* < 0.05)

In adults, the heritability of body composition ranged from 0.28–0.50, with waist circumference and body fat mass showing the lowest and highest, respectively (Table 3). When adjusting for shared environment, the heritability of fat free mass was non-significant. Measures of insulin secretion and sensitivity had heritability estimates between 0.25 and 0.37 (Table 3).

### Genetic correlations between birth weight and insulin sensitivity

Subsequently, genetic correlations between traits having a significant genetic heritability were calculated, and we observed a high genetic correlation between birth weight and ponderal index (ρG = 0.88 (95% CI: 0.76; 1.00), *p* = 3.0*10^− 10^). Yet it was still significantly different from 1 (*p* = 0.007). We therefore continued with separate genetic correlations for birth weight and ponderal index, as the genetics of these traits are not identical.

When we proceeded to assess the genetic correlations between birth weight and ponderal index and insulin sensitivity, both birth weight and ponderal index showed significant positive genetic correlations with the OGTT-derived Matsuda insulin sensitivity index (ρG = 0.36 (95% CI: 0.03; 0.69), *p* = 0.047), (ρG = 0.52 (95% CI: 0.15; 0.89), *p* = 0.02), respectively (Table 4).

**Table 4:** Correlations between measures of birth body composition and adult measures of insulin sensitivity

	Birth weight (g)			Ponderal index		
	*n*	ρP	ρG (95% CI)	*n*	ρP	ρG (95% CI)
Insulin sensitivity in adulthood						
HOMA-IR	376	- 0.12	- 0.31 (- 0.64; 0.02)	376	- 0.09	- 0.35 (- 0,76; 0.06)
Matsuda insulin sensitivity index	376	0.15*	0.36 (0.03; 0.69)*#	376	0.14*	0.52 (0.15; 0.89)*#

Phenotypic correlations between traits were estimated as ρP, and genetic correlations were estimated as ρG (95% CI). Correlations were estimated in non-diabetic relatives of patients with type 2 diabetes mellitus. All correlations were adjusted for sex. *Correlation significantly different from zero (*p* < 0.05). #Correlation remained significant following adjustment for both birth weight and adult type 2 diabetes susceptibility SNPs. All genetic correlations were significantly different from 1 (*p* < 0.05)

### Genetic correlations between birth weight and insulin secretion

Ponderal index showed an inverse genetic correlation with AUC for insulin (ρG = − 0.66 (95% CI: -1.13; -0.19), *p* = 0.01) (Table 5), however, this correlation was not significantly different from 1 (*p* > 0.05). Ponderal index also showed an inverse genetic correlation with fasting insulin (ρG = − 0.47 (95% CI: -0.86; − 0.08), *p* = 0.029), yet, this correlation did not withstand correction for multiple testing.

**Table 5:** Correlations between measures of birth body composition and adult measures of insulin secretion

	Birth weight (g)			Ponderal index		
	*n*	ρP	ρG (95% CI)	*n*	ρP	ρG (95% CI)
Insulin secretion in adulthood						
HOMA-B (%)	376	- 0.01	- 0.22 (- 0.57; 0.13)	376	- 0.08	- 0.33 (- 0.76; 0.10)
BIGTT-AIR	349	0.11	- 0.03 (- 0.44; 0.38)	349	0.03	- 0.10 (- 0.61; 0.41)
Insulinogenic index	348	0.06	0.08 (- 0.31; 0.47)	348	- 0.02	- 0.18 (- 0.67; 0.31)
AUC insulin 4 h (pmol/l*min)	345	- 0.14*	- 0.29 (- 0.72; 0.14)	345	- 0.14*	- 0.66 (- 1.13; - 0.19)*#
Fasting serum insulin (pmol/l)	377	- 0.09	- 0.28 (- 0.61; 0.05)	377	- 0.13*	- 0.47 (- 0.86;− 0.08)*#

Phenotypic correlations between traits were estimated as ρP, and genetic correlations were estimated as ρG (95% CI). Correlations were estimated in non-diabetic relatives of patients with type 2 diabetes mellitus. All correlations were adjusted for sex. *Correlation significantly different from zero (*p* < 0.05). #Correlation remained significant following adjustment for both birth weight and adult type 2 diabetes susceptibility SNPs

AUC: area under the curve, BIGTT-AIR: index for acute insulin response, HOMA-B: HOMA for beta cell function

### Genetic correlations between birth weight and adult body weight

When assessing measures of body composition at birth and in adulthood (Table 6), a significant correlation between birth weight and adult weight was observed (ρG = 0.38 (95% CI: 0.09; 0.67), *p* = 0.02) (Table 6). Unfortunately, information on gestational age was unavailable, so we performed a sensitivity analysis by only including individuals having a birth weight > 2500 g, thereby likely excluding individuals born premature. This did, however, not affect our findings, as we found a significant genetic correlation between birth weight and adult weight (ρG = 0.42 (95% CI: 0.13; 0.71), *p* = 0.01).

**Table 6:** Phenotypic and genetic correlations between measures of body composition at birth and in adulthood

	Birth weight (g)			Ponderal index		
Traits measured in adulthood	*n*	ρP	ρG (95% CI)	*n*	ρP	ρG (95% CI)
Weight (kg)	411	0.25*	0.38 (0.09; 0.67)*#	411	0.15	0.12 (- 0.31; 0.55)
Height (cm)	411	0.16*	0.17 (- 0.14; 0.48)	411	- 0.08	0.33 (- 0.02; 0.68)
Waist (cm)	406	0.08	0.08 (- 0.27; 0.43)	406	0.08	- 0.24 (- 0.65; 0.17)
Waist-hip ratio	406	- 0.03	0.03 (- 0.32; 0.38)	406	- 0.01	- 0.36 (- 0.75; 0.03)
BMI (kg/m^2^)	411	0.16*	0.24 (- 0.07; 0.55)	411	0.12	- 0.01 (- 0.42; 0.40)
Fat mass (kg)	376	0.04	0.04 (- 0.29; 0.37)	376	0.01	- 0.06 (- 0.47; 0.35)

Phenotypic correlations between traits were estimated as ρP (95% CI), and genetic correlations were estimated as ρG (95% CI). All correlations were adjusted for sex. *Correlation significantly different from zero (*p* < 0.05). #Correlation remained significant following adjustment for both birth weight and adult BMI susceptibility SNPs. All genetic correlations were significantly different from 1 (*p* < 0.05)

### Genetic associations

To estimate the genetic overlap ascribed to variants known to influence the analysed trait, the statistically significant genetic correlations between birth weight and adult glycaemic regulation were adjusted for birth weight and T2DM susceptibility SNPs (Additional file 1). Likewise, significant genetic correlations between birth weight and adult body weight were adjusted for high effect-sized birth weight and BMI susceptibility SNPs. Following this adjustment, all significant genetic correlations remained unaffected (Table 4, Table 5 and Table 6).

## Discussion

### Birth weight is highly heritable

The highest heritability for body-composition at birth was observed for birth weight (0.80). The heritability of birth weight has in twin studies previously been found to be between 0.15–0.40 [26–28], however, such studies may be affected by competition for nutrients in the placenta. In contrast, a large study of offspring of twin-pairs (*n* > 5000) found a heritability of birth weight of 0.70 [29], and a heritability of 0.92 has been reported in a Hispanic family-based study [30]. To date, only one previous study, comprising 917 individuals, has investigated the heritability of ponderal index which was found to be 0.39. The same study also reported the heritability for birth weight to be 0.67 [31]. In studies of twins and singletons, the heritability of birth length has previously been reported to be 0.27 and 0.26, respectively [32]. However, in the current study, the genetic heritability for birth length was not significantly different from 0. Potentially, this could be due to the low statistical power in our study or due to the fact that shared environmental effect was taken into account.

Choh and colleagues also tested whether the genetics involved in ponderal index are overlapping with the genetics of birth weight, and the genetic correlation between these traits were 0.30 (SE: 0.12) [31]. In the present study, the genetic overlap between birth weight and ponderal index was 0.88, yet, still significantly different from 1. Thus, birth weight and ponderal index are, at least in part, genetically distinct.

### Birth weight and insulin secretion/sensitivity in adulthood have overlapping genetic pathways

Our primary aim was to investigate the genetic overlap between fetal body composition and measures of adult insulin sensitivity. We found a positive correlation both between birth weight and ponderal index and Matsuda index of insulin sensitivity. These correlations indicate, that genetic variation having a diminishing effect on fetal body composition also decrease insulin sensitivity in support of the fetal insulin hypothesis, suggesting that a genetic disposition to insulin resistance is affecting not only adult metabolism but also fetal growth. Although not statistically significant, both birth weight and ponderal index displayed negative directionally genetic correlations with HOMA-IR, further supporting the fetal insulin hypothesis.

When assessing the genetic correlations between fetal body composition and measures of insulin secretion, we observe negative correlations between ponderal index and insulin secretion. This is implying that a low ponderal index is linked to an increased insulin secretion in adulthood, which could be a compensatory mechanism in response to insulin resistance, thus, it is also in accordance with the fetal insulin hypothesis. This genetic correlation was not observed between birth weight and indices of insulin secretion. With insulin being an important intra-uterine growth factor, a reduction in ponderal index due to an increased birth length may be a result of higher levels of fetal insulin.

### Birth weight and adult weight share genetic pathways

A positive genetic correlation was also found between birth weight and adult weight, yet, no significant genetic correlation was found between birth weight and adult fat mass. Thus, we speculate that the genetic overlap between birth weight and adult weight may be mediated through regulation of lean body mass. Body composition differentiates between men and women, and although our analyses were adjusted for sex, sex-specific genetic correlations between birth weight and adult measures of body composition would have been desirable. Unfortunately, our study sample did not allow us to perform sex-specific analyses, and larger studies are required to further investigate this hypothesis.

Cross-trait LD score regression has been used to estimate genetic overlap between several traits [14, 33]. Using this approach, Bulik-Sullivan et al. analysed data from 24 GWASs comprising > 1,5 million individuals, and although no significant genetic correlations between birth weight and overweight or obesity were found, the authors identified a positive genetic correlation between birth weight and adult waist circumference (ρG = 0.14 (SE: 0.06)) [33]. A succeeding study combining data from 37 GWAS and encompassing up to 153,781 individuals also identified a genetic correlation between birth weight and adult BMI (ρG = 0.11 (SE: 0.03)) [14]. However, traits differentiating between lean and fat body mass were not investigated in either studies, nor was the effect of rare genetic variation accounted for as these studies were based on GWAS data.

In the current study, individual or combined adjustment for SNPs with a known substantial effect size on either birth weight, BMI or T2DM did not alter the genetic correlations. Thus, none of the 45 included SNPs therefore significantly influcences the identified genetic correlations between body composition at birth and adult measures of insulin secretion and sensitivity or between body composition at birth and in adulthood. The lack of effect of BMI SNPs on both fetal and adult body composition maybe not be surprising considering that it has previously been established that GWAS-identified BMI loci only affect BMI at certain life stages [34]. Interestingly, Horikoshi and colleagues estimated that 0.39 (95% CI: 0.17–0.61) of the co-variance between birth weight and adult BMI could be explained by the > 300,000 SNPs directly genotyped in their study [14], further demonstrating that the shared genetics between birth weight and adult BMI can be attributed to the effect of a vast number of common genetic variations rather than to a few high effect sized variants.

Finally, it should be noted that all the genetic correlations should be considered relative to the genetic heritability of the examined traits, thus, a large genetic correlation in the light of low heritability indicates, that only a minor proportion of the phenotypic variation is explained by overlapping genetics.

The present study is limited by the relatively small sample size, which reduces our statistical power. We can therefore not exclude the possibility that non-significant genetic correlations are a result of type 2 statistical errors. Furthermore, although the measurements of fetal body composition were obtained by trained professional staff, especially measurements of birth length are known to be prone for measuring error. As ponderal index is a function of birth weight and birth length, potential measuring errors may affect our findings and could explain some of the observed differences between the results obtained for birth weight and ponderal index. In addition, the family design may inflate our measures of heritability as compared to the more recent approach of estimating heritability by genotyping common genetic variants in large population-based populations. Although such an approach is statistically powerful, it does not include the effect of variants not captured by such genotyping, including rare variants. Due to the large samples often employed in such analysis, this approach does not enable the inclusion of detailed phenotypes, e.g. OGTT-derived measures. This in in contrast to our family-based study, which includes detailed phenotypes related to body composition, insulin secretion and insulin sensitivity in adulthood and takes both common and rare forms of genetic variation as well as shared environment into account.

## Conclusions

Among Danes of Caucasian ethnicity, the genetics of birth weight and ponderal index appear to be partly shared with the genetics of adult insulin resistance, supporting the fetal insulin hypothesis. Yet, only ponderal index shared genetic regulation with adult insulin release and only birth weight shared with adult weight. These genetic correlations could not be explained by variants known to influence any of the above-mentioned traits.

## Additional files

Additional file 1: Genotyped birth weight, BMI and T2DM-associated SNPs included in analyses. Effect sizes are presented as β-values for birth weight (in grams) and adult BMI (change in BMI), or as an OR for adult risk of T2DM. SNP: single nucleotide polymorphism, T2DM: type 2 diabetes mellitus. (DOCX 580 kb)

Additional file 2: Calculation of insulin secretion indices. BIGTT-AIR: index for acute insulin response, BMI: body mass index, HOMA-B: HOMA for beta cell function, HOMA-IR: HOMA for insulin resistance. (DOCX 12 kb)

## Abbreviations

AUC: Area under the curve
BIGTT-AIR: Index for acute insulin response
BMI: Body mass index
GWAS: Genome-wide association study
HOMA-B: HOMA for beta cell function
HOMA-IR: HOMA for insulin resistance
LD: Linkage disequilibrium
OGTT: Oral glucose tolerance test
SE: Standard error
SNP: Single nucleotide polymorphism
T2DM: Type 2 diabetes mellitus
ρE: Environmental correlation
ρG: Genetic correlation
ρP: Phenotypic correlation
