# Supplementary material for: Genetic insights into fetal growth and measures of glycaemic regulation and adiposity in adulthood: a family-based study
Source: BMC Med Genet. 2018 Dec 4;19:207. doi: 10.1186/s12881-018-0718-2 (PMC6278142; doi:10.1186/s12881-018-0718-2)
Supplement: Supplementary file 1 — Genotyped birth weight, BMI and T2DM-associated SNPs included in analyses. Effect sizes are presented as β-values for birth weight (in grams) and adult BMI (change in BMI), or as an OR for adult risk of T2DM. SNP: single nucleotide polymorphism, T2DM: type 2 diabetes mellitus. (DOCX 580 kb) [file 12881_2018_718_MOESM1_ESM.docx]

| Associated trait | SNP id | Nearest gene(s) | Alleles  (effect/other) | Effect | | Reference |
| --- | --- | --- | --- | --- | --- | --- |
|  |  |  |  | *β* | OR |  |
| Birth weight | rs138715366 | *YKT6-GCK* | C/T | 0.24 | - | [1] |
|  | rs144843919 | *SUZ12P1-CRLF3* | G/A | 0.066 | - | [1] |
|  | rs28510415 | *PTCH1* | G/A | 0.056 | - | [1] |
|  | rs13322435 | *CCNL1-LEKR1* | A/G | 0.053 | - | [1] |
|  | rs62466330 | *MLXIPL* | C/T | 0.049 | - | [1] |
|  | rs72851023 | *INS-IGF2* | T/C | 0.048 | - | [1] |
|  | rs62240962 | *SREBF2* | C/T | 0.047 | - | [1] |
|  | rs1374204 | *EPAS1* | T/C | 0.047 | - | [1] |
|  | rs11719201 | *ADCY5* | T/C | 0.046 | - | [1] |
|  | rs1351394 | *HMGA2* | T/C | 0.044 | - | [1] |
|  | rs35261542 | *CDKAL1* | C/A | 0.044 | - | [1] |
|  | rs7964361 | *IGF1* | A/G | 0.039 | - | [1] |
|  | rs7575873 | *ATAD2B* | A/G | 0.038 | - | [1] |
|  | rs74233809 | *NT5C2* | C/T | 0.037 | - | [1] |
|  | rs7076938 | *ADRB1* | T/C | 0.036 | - | [1] |
| Adult BMI | rs1558902 | *FTO* | A/T | 0.082 | - | [2] |
|  | rs17024393 | *GNAT2; AMPD2* | C/T | 0.066 | - | [2] |
|  | rs13021737 | *TMEM18* | G/A | 0.060 | - | [2] |
|  | rs6567160 | *MC4R* | C/T | 0.056 | - | [2] |
|  | rs11847697 | *PRKD1* | T/C | 0.049 | - | [2] |
|  | rs543874 | *SEC16B* | G/A | 0.048 | - | [2] |
|  | rs13107325 | *SLC39A8* | T/C | 0.048 | - | [2] |
|  | rs16851483 | *RASA2* | T/G | 0.048 | - | [2] |
|  | rs2207139 | *TFAP2B* | G/A | 0.045 | - | [2] |
|  | rs1516725 | *E7V5* | C/T | 0.045 | - | [2] |
|  | rs11030104 | *BDNF* | A/G | 0.041 | - | [2] |
|  | rs12446632 | *GPRC5B; IQCK* | G/A | 0.040 | - | [2] |
|  | rs10938397 | *GNPDA2; GABRG1* | G/A | 0.040 | - | [2] |
|  | rs2287019 | *QPCTL; GIPR* | C/T | 0.036 | - | [2] |
|  | rs3101336 | *NEGR1* | C/T | 0.033 | - | [2] |
| Adult risk of T2DM | rs7903146 | *TCF7L2* | T/C | - | 1.37 | [3-5] |
|  | rs5945326 | *DUSP9* | A/G | - | 1.27 | [6] |
|  | rs10811661 | *CDKN2A; CDKN2B* | T/C | - | 1.20 | [5, 7, 8] |
|  | rs2943641 | *IRS1* | T/C | - | 1.19 | [9] |
|  | rs9939609 | *FTO* | A/T | - | 1.17 | [5, 8, 10] |
|  | rs7578597 | *THADA* | T/C | - | 1.15 | [11] |
|  | rs1111875 | *HHEX; IDE* | C/T | - | 1.15 | [4, 7, 8] |
|  | rs3802177 | *SLC30A8* | G/A | - | 1.15 | [6] |
|  | rs10946398 | *CDKAL1* | C/A | - | 1.14 | [5, 7, 8, 12] |
|  | rs5215 | *KCNJ11* | C/T | - | 1.14 | [5, 7] |
|  | rs4402960 | *IGF2BP2* | T/G | - | 1.14 | [5, 7, 8] |
|  | rs1552224 | *CENTD2* | A/C | - | 1.14 | [6] |
|  | rs1801282 | *PPARG* | C/G | - | 1.14 | [5, 8] |
|  | rs10923931 | *NOTCH2* | T/G | - | 1.13 | [11] |
|  | rs10401969 | *CILP2* | C/T | - | 1.13 | [13] |

References

[1] Horikoshi M, Beaumont RN, Day FR, et al. (2016) Genome-wide associations for birth weight and correlations with adult disease. Nature 538: 248-252

[2] Locke AE, Kahali B, Berndt SI, et al. (2015) Genetic studies of body mass index yield new insights for obesity biology. Nature 518: 197-206

[3] Grant SF, Thorleifsson G, Reynisdottir I, et al. (2006) Variant of transcription factor 7-like 2 (TCF7L2) gene confers risk of type 2 diabetes. Nat Genet 38: 320-323

[4] Sladek R, Rocheleau G, Rung J, et al. (2007) A genome-wide association study identifies novel risk loci for type 2 diabetes. Nature 445: 881-885

[5] Scott LJ, Mohlke KL, Bonnycastle LL, et al. (2007) A genome-wide association study of type 2 diabetes in Finns detects multiple susceptibility variants. Science 316: 1341-1345

[6] Voight BF, Scott LJ, Steinthorsdottir V, et al. (2010) Twelve type 2 diabetes susceptibility loci identified through large-scale association analysis. Nat Genet 42: 579-589

[7] Saxena R, Voight BF, Lyssenko V, et al. (2007) Genome-wide association analysis identifies loci for type 2 diabetes and triglyceride levels. Science 316: 1331-1336

[8] Zeggini E, Weedon MN, Lindgren CM, et al. (2007) Replication of genome-wide association signals in UK samples reveals risk loci for type 2 diabetes. Science 316: 1336-1341

[9] Rung J, Cauchi S, Albrechtsen A, et al. (2009) Genetic variant near IRS1 is associated with type 2 diabetes, insulin resistance and hyperinsulinemia. Nat Genet 41: 1110-1115

[10] Frayling TM, Timpson NJ, Weedon MN, et al. (2007) A common variant in the FTO gene is associated with body mass index and predisposes to childhood and adult obesity. Science 316: 889-894

[11] Zeggini E, Scott LJ, Saxena R, et al. (2008) Meta-analysis of genome-wide association data and large-scale replication identifies additional susceptibility loci for type 2 diabetes. Nat Genet 40: 638-645

[12] Steinthorsdottir V, Thorleifsson G, Reynisdottir I, et al. (2007) A variant in CDKAL1 influences insulin response and risk of type 2 diabetes. Nat Genet 39: 770-775

[13] Morris AP, Voight BF, Teslovich TM, et al. (2012) Large-scale association analysis provides insights into the genetic architecture and pathophysiology of type 2 diabetes. Nat Genet 44: 981-990
