# Supplementary material for: Genetic insights into fetal growth and measures of glycaemic regulation and adiposity in adulthood: a family-based study
Source: BMC Med Genet. 2018 Dec 4;19:207. doi: 10.1186/s12881-018-0718-2 (PMC6278142; doi:10.1186/s12881-018-0718-2)
Supplement: Supplementary file 2 — Calculation of insulin secretion indices. BIGTT-AIR: index for acute insulin response, BMI: body mass index, HOMA-B: HOMA for beta cell function, HOMA-IR: HOMA for insulin resistance. (DOCX 12 kb) [file 12881_2018_718_MOESM2_ESM.docx]

| Trait | Calculation |
| --- | --- |
| HOMA-B | (20*fasting insulin) / (fasting glucose – 3.5) |
| HOMA-IR | fasting glucose * fasting insulin / 22.5 |
| BIGTT-AIR | exp(4.90-(0.00402*fasting insulin) - (0.000556*insulin(30 minutes)) - (0.00127*insulin(120 minutes)) - (0.152*fasting glucose) - (0.00871*glucose(30 minutes)) - (0.0373*glucose(120 minutes)) - (0.145*sex) - (0.0376*BMI))) |
| The insulin sensitivity Matsuda index | 10000/(√((fasting glucose*18)*(fasting insulin/6.945)*(mean glucose 0, 30, 120 minutes)*18)*(mean insulin 0, 30, 120 minutes/6.945))) |
| Insulinogenic index | (insulin 30 minutes - fasting insulin) / (glucose 30 minutes - fasting glucose) |
